# Impact of Prior COVID-19 Immunization and/or Prior Infection on Immune Responses and Clinical Outcomes

**DOI:** 10.3390/v16050685

**Published:** 2024-04-26

**Authors:** Achilleas Livieratos, Charalambos Gogos, Karolina Akinosoglou

**Affiliations:** 1 Independent Researcher, 15238 Athens, Greece; 2 Department of Medicine, University of Patras, 26504 Rio, Greece; cgogos@med.upatras.gr (C.G.); akin@upatras.gr (K.A.); 3 Department of Internal Medicine and Infectious Diseases, University General Hospital of Patras, 26504 Rio, Greece

**Keywords:** SARS-CoV-2 infection, immune responses, humoral immunity, transmission, vaccination, cellular immunity, clinical outcomes, COVID-19

## Abstract

Cellular and humoral immunity exhibit dynamic adaptation to the mutating SARS-CoV-2 virus. It is noteworthy that immune responses differ significantly, influenced by whether a patient has received vaccination or whether there is co-occurrence of naturally acquired and vaccine-induced immunity, known as hybrid immunity. The different immune reactions, conditional on vaccination status and the viral variant involved, bear implications for inflammatory responses, patient outcomes, pathogen transmission rates, and lingering post-COVID conditions. Considering these developments, we have performed a review of recently published literature, aiming to disentangle the intricate relationships among immunological profiles, transmission, the long-term health effects post-COVID infection poses, and the resultant clinical manifestations. This investigation is directed toward understanding the variability in the longevity and potency of cellular and humoral immune responses elicited by immunization and hybrid infection.

## 1. Introduction

Following the appearance of Severe Acute Respiratory Syndrome Coronavirus 2 (SARS-CoV-2), multiple successive waves of infections have been observed from different strains [1]. Until April 2024, the virus had impacted over 770 million people globally, resulting in 7.0 million deaths [2]. Multiple risk factors such as advanced age, underlying health conditions, and a history of pneumonia have been linked to COVID-19 susceptibility [3]. Similar to other viral infections, it has been demonstrated that individuals can mobilize their innate and adaptive immunity against COVID-19, facilitating its clearance and impeding its spread [4].

The development of memory B and T cells, induced by either infection or vaccination, is pivotal for a robust immune reaction in the form of antibody and cellular responses [5,6,7,8,9]. Associations between antibody levels and infection susceptibility have been documented, while monitoring humoral response levels could serve as indicator of immune protection [10,11,12]. Research has indicated prolonged virus-specific cellular immunity in previously infected individuals, persisting for up to 8 months post-infection [4,13,14,15]. In those who recovered from the original SARS-CoV, cellular immune responses remained detectable for up to two decades, while memory B cells and antibodies were generally undetectable after that period [4,16]. However, the continuing effectiveness of humoral and cellular immunity memory in recovered individuals is not yet fully understood.

Vaccinations have been shown to reduce the severity of the disease but may not entirely prevent infection [1,17,18]. There is a strong association between neutralizing antibody concentrations and immunization efficacy [19]. Although vaccines decrease the occurrence of clinically severe outcomes, such as hospitalization and mortality, protection declines over time [18]. The immunity provided by a two-dose vaccine remains significant against severe outcomes for approximately 5–6 months [20]. The decline in immunity can compromise host defenses. Notably, a third vaccine dose has been linked to a substantial increase in immunity [21,22,23]. Recent findings indicate that a fourth dose can stimulate an enhanced immunologic response in individuals previously vaccinated with three doses, regardless of the initial vaccine type used [24]. With more individuals contracting the virus globally, post-infection vaccination is expected to increase [25,26,27,28,29,30]. Prior SARS-CoV-2 infection appears to enhance vaccine-induced immune responses, but the long-term implications are not clear.

Elevated levels of specific inflammatory biomarkers, including interferon-γ, are directly involved in the humoral immunity response [31,32]. Furthermore, higher concentrations of pro-inflammatory cytokines following vaccination are associated with heightened antibody responses among blood donors and organ transplant recipients [31,33]. Cytokines and chemokines are critical to the body’s response to infections and vaccines [31]. However, our understanding of how COVID-19 vaccination affects cytokine and chemokine levels in the short or long term, as well as their trajectories in symptomatic COVID-19 patients, is limited.

Studies have reported suboptimal vaccine-induced immune responses in patient populations with chronic conditions and those undergoing immunosuppressive treatment [34,35,36,37,38,39,40,41,42,43,44,45,46,47]. Comprehensive population analyses, including immunocompromised participants, have revealed reduced seropositivity for SARS-CoV-2 spike protein antibodies after vaccination and only modest vaccine efficacy [48,49]. Additionally, being immunocompromised after immunization may increase the risk for severe clinical outcomes [50].

This work aims to methodically examine the recent literature to assess the impact of previous immunization or infection with COVID-19 on subsequent immune responses and clinical outcomes.

## 2. Materials and Methods

**Figure 1:**
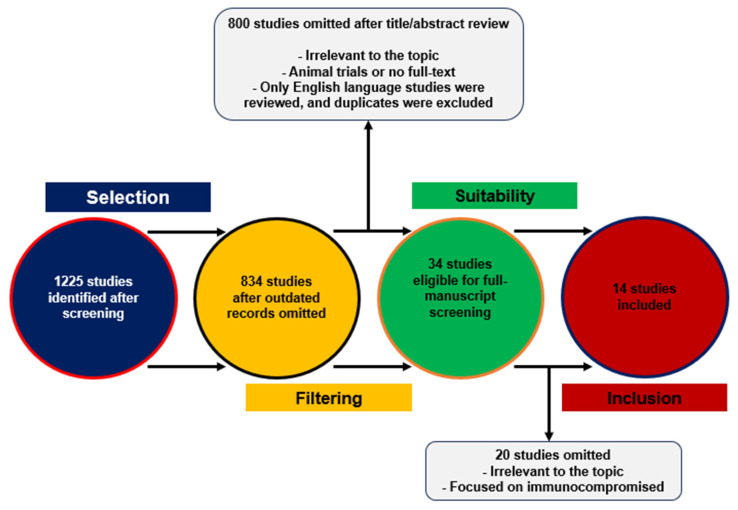
Schematic for study selection.

During the literature search, conducted from January 2021 until March 2024 using the PubMed database, 1225 articles were initially identified as potentially relevant. The key words applied included SARS-CoV-2 infection, long COVID, immune response, inflammatory, vaccination, symptoms, and transmission. Specifically, the search strategy included the following query string: SARS-CoV-2 infection AND vaccination AND immunity AND symptoms AND Long COVID. Expanding the query string further resulted in a restricted article pool. Subsequently, 391 studies were considered obsolete and omitted from consideration. Following a close review of these 834 studies, 800 were eliminated due to irrelevant subject matter, animal experiments, or studies prior to 2021. Only English-language papers were reviewed, and duplicates were excluded. Further rigorous assessments of the full texts of the remaining 34 studies led to the exclusion of an additional 20 studies. Consequently, 14 studies were ultimately deemed appropriate and included in the systematic review, strictly adhering to the study topic. Two independent researchers reviewed the articles and hand-searched literature. Disagreements were discussed and resolved. A graphical summary of the literature retrieval flow is presented in Figure 1.

Among the 14 articles analyzed, most studies (*n* = 3) were carried out in the United States of America. The remaining studies originated from China, Thailand, Canada, Sweden, Switzerland, the United Kingdom, Spain, the Republic of Korea, and Qatar. Reinfection was established through a second positive test result. This second positive result had to be recorded at least 3 months after the initial diagnosis. These research articles explored various aspects of SARS-CoV-2 reinfection, including its severity and subsequent health implications, the related humoral and cell-mediated immune responses, and the long-term effects following the infection.

Specifically, as illustrated in Table 1, the 14 included studies varied in terms of population sizes, immunization status, and research aims. These studies included a mixture of unvaccinated, partly vaccinated, and fully vaccinated cohorts. Two of the included studies were network meta-analyses, with a particular focus on transmission and clinical outcomes. Collectively, these articles aimed to capture the heterogeneity of infection-experienced/naïve and immunization-experienced/naïve cohorts and establish an association with inflammatory responses, the durability of the adaptive immune response, and clinical outcomes.

## 3. Discussion

### 3.1. Immune Responses

Research has examined the endurance of immune responses in individuals who have recovered from illness. Cohen et al.’s research revealed enduring and robust antibody, memory T cell, and B cell levels for approximately eight months after the initial infection [51]. Li et al. reported that, a year after diagnosis, over 70% of individuals still tested positive for IgG antibodies [52]. However, T cell reactions as well as the efficacy of circulating antibodies to various strains were not measured in that study. Rank et al. found that about two-thirds of the study subjects showed persistent IFNγ-specific T cell responses at the end of a 12-month period [53]. More recent findings from Zhang et al. suggest that both neutralizing antibody and memory T cell immunity remains robust for a year following diagnosis of the disease, with human peripheral blood mononuclear cells (PBMCs) exhibiting sustained activity for nine days ex vivo [54]. However, the scope of Zhang and colleagues’ study did not extend to evaluating the responses of humoral or cellular immunity to different viral strains [54]. Thus, among healthy controls, recovery from a natural infection appears to provide robust immune protection against reinfection.

#### 3.1.1. Cellular Immunity

The pivotal role of the adaptive immune response in determining clinical outcomes following a viral infection, including the impact on immunization, is well documented [55]. T cell responses, linked to early-stage protective immunity, emerge early in infection. However, these responses diminish in severe cases of the disease, characterized by significant activation and reduced lymphocyte numbers [55]. Evidence suggests that a T cell subset, initially activated by exposure to seasonal coronaviruses, exhibits cross-reactivity to SARS-CoV-2, potentially enhancing clinical protection, especially in younger populations [55]. T cell memory includes widespread viral protein recognition, with individuals’ immune systems recognizing around 30 distinct epitopes [55]. This lasting recognition could play a crucial role in mitigating the impact of new viral mutations, providing a foundation for sustained protection against severe illness from future variants. Immunization leads to robust T cell immunity, thus contributing significantly to preventing severe disease outcomes, hospitalizations, and fatalities [55]. New and combined vaccine approaches may offer opportunities to further enhance these cellular immune reactions. Thus, T cell immunity appears critical in managing viral infection, despite its significance being previously underestimated.

Research indicates that T cell immunity to SARS-CoV-2 declines at a slower rate than neutralizing antibody levels over time [4]. Memory T cells from the SARS-CoV epidemic have been detectable up to 17 years post-infection, showing notable cross-variant effectiveness [56]. Interestingly, there is evidence that T cell memory correlates with the reduced severity of influenza in the absence of neutralizing antibodies [57]. Longitudinal assessments of antibody and T cell immunity to primary infection over 20 months revealed that more than 94% of participants seroconverted for IgG specific to Nucleocapsid (N) and Spike (S) proteins one-month post-infection [58]. Additionally, the majority of participants showed both non-S- and S-specific T cell detection, indicating an adaptive immune response to the infection [58]. The study observed that while the detectability of non-S-specific humoral and cellular immunity reduced over the 20-month period, it was still observable in most participants, implying the development of efficient long-term immune memory [59,60]. When examining responses to non-S antigens specifically, a greater proportion of participants maintained measurable T cell responses as opposed to circulating antibody levels [59,60]. This supports the notion of enduring cellular immune memory akin to observations from the initial SARS-CoV outbreak. The humoral and cellular immune responses specific to the S-protein also declined within the first ten months after infection; however, vaccination subsequently induced a strong S-specific memory response [58]. These results corroborate those of other longitudinal studies that demonstrate waning antibody and T cell immunity after 12 months, while the potentiation of an S-specific memory response may be accomplished by subsequent immunization [61,62]. The analysis of both S- and non-S-specific T memory cells differs between immunity arising from a prior infection or a subsequent immunization [63]. Gittelman et al. investigated the specificity toward S and other targets and reported an increased presence of T cell receptors 15 months after COVID-19 infection, and an even further increase in clonality and diversity for the S protein in vaccinated participants, in contrast to non-S proteins [63]. These data validate long-term cellular response findings highlighted by Gittelman et al., distinguishing the immunity profiles following a primary infection versus those augmented by vaccination [63]. The results consistently point to the natural persistence of generic immunity for approximately 20 months [63]. Despite the observed waning in viral-specific immunity, a non-S immune response was still detectable even after 20 months [63].

#### 3.1.2. Humoral Immunity

Recent findings indicate that individuals who had prior natural infection followed by two to four vaccine doses exhibited a heightened and more enduring response of Ig anti-RBD (Receptor Binding Domain) antibodies over a year compared to those who were solely vaccinated. This observation aligns with previous research that suggested that individuals who experienced a breakthrough infection after receiving two doses of the CoronaVac vaccine showed elevated Ig anti-RBD levels compared to those participants who received a third dose of AZD1222, though they were similar to the antibody levels found following a third dose of the BNT162b2 booster [64,65,66,67]. Additionally, a comprehensive study in Monaco demonstrated that hybrid immunity generates potent humoral immunity against infection [68].

Equally, a previously published study from Denmark was conducted in fully vaccinated participants with BNT162b2 with/without an earlier infection [69]. This work reported robust hybrid immunity resulting from high antibody levels in the previously infected cohort (72%) versus the non-infected participants (35%) [69]. Moreover, research from a substantial Swedish cohort found that hybrid immunity provided marked protection against COVID-19 reinfection and hospitalization [69].

It has been established that the Omicron variant causes less severe infections with a mortality rate substantially lower by 6.2 times than that of Delta variant infections [70]. However, Omicron variants are more effective at evading the immune responses prompted by vaccination [71,72]. The efficacy of the two-dose vaccine, based on the original Wuhan strain, is reduced against the Omicron variant, with a noted rapid decline in the vaccine-induced humoral immunity [72,73,74,75,76]. Consequently, a third booster dose is mandatory to reinforce immunity and provide further protection against severe clinical outcomes, particularly in high-risk individuals [21,22,23].

Additional studies comparing humoral immunity in three-dose versus two-dose vaccinations across individuals who have not encountered infection previously also indicated improved antibody longevity post the three-dose regimen [68]. Despite the evasive nature of the Omicron variant and its various subtypes, immunization boosters and hybrid immunity result in strong humoral responses in healthy controls. Naturally, elderly and immunocompromised patients continue to be at an increased risk as humoral immunity is expected to wane faster in these individuals versus healthy controls.

#### 3.1.3. Systematic Inflammatory Response

Recent studies have shown that fully immunized individuals exhibited milder cytokine inflammatory responses systemically after infection compared to those who were not vaccinated, both in the early and late stages of infection [31]. As individuals recovered from symptomatic COVID-19, all vaccination statuses were associated with a decrease in these inflammatory markers [31]. Although unvaccinated individuals showed, longitudinally, a more rapid reduction in these markers, the average levels of systemic cytokines, such as interleukin-7 (IL-7), remained elevated compared to the levels in vaccinated individuals three months post-diagnosis due to initially higher inflammatory levels [31]. These findings suggest that vaccination has a mitigating effect on inflammation, even in cases of symptomatic breakthrough infections. Research indicates that vaccinated individuals who experience breakthrough infections demonstrate superior cellular immune responses, leading to decreased inflammation compared to their unvaccinated counterparts [31]. Other studies have linked increased cytokine and chemokine levels with greater disease severity in SARS-CoV-2 infections and have noted associations of heightened IL-7 with chronic inflammatory conditions, IL-8 with lung hyperinflammation and prolonged disease in severe COVID-19 cases, and overrepresented VEGF-A in the pulmonary tissue of COVID-19 fatalities [77,78,79,80]. Notably, since post-infection unvaccinated participants demonstrated elevated levels of systemic inflammatory markers compared to their immunized peers, it is understood that prolonged inflammation resolution occurs in unvaccinated individuals [31].

Systematic reviews have identified a correlation between vaccination and a reduced likelihood of experiencing post-COVID-19 symptoms [81]. The prolonged elevation of cytokines in unvaccinated individuals may partially explain the increased propensity for post-COVID-19 conditions in certain cases [81]. Comparative studies of older vaccinated individuals have showed a 40% lower level of IL-22 during recovery compared to unvaccinated individuals [82]. In the younger demographic, the influence of vaccination was less pronounced, with age-related variations in IL-22 expression suggested as a possible explanation [82].

Individuals who received both adenovirus vector and mRNA vaccines exhibited similar cytokine levels, although mRNA vaccines may further reduce IL-8 and VEGF-A [31]. There have been assumptions that components of the adenovirus vaccine may exacerbate inflammation, but data remain insufficient regarding the interplay between vaccination types and cytokine profiles in symptomatic COVID-19 patients. Consequently, further research is needed to establish a direct correlation between cytokine profiles and vaccine type.

### 3.2. Clinical Outcomes

The Omicron variant demonstrated reduced susceptibility to protection from immunization and prior SARS-CoV-2 infection compared to the Delta variant, but hybrid immunity still provided considerable protection against critical outcomes requiring intensive care unit (ICU) admissions and mortality [75].

Booster doses significantly improve immunization protection against several clinical outcomes. However, their effect diminishes notably over time. Additionally, monoclonal antibody treatment administered post-Omicron infection has been found to significantly decrease the possibility of hospitalization and mortality [75].

The milder clinical outcomes reported with the Omicron strain compared to those of earlier variants can be attributed to the reduced virulence of Omicron and increased population immunity due to booster vaccinations and past infections [71,72]. Two factors contributed to this; firstly, during the early days of the Omicron wave, many of the previous infections were due to the Delta strain of the virus leading to weaker immunological responses. Secondly, during the Omicron wave, individuals experienced a re-infectivity delay before positive cases spiked. As a result, the Delta wave patients had more recently experienced a previous COVID-19 diagnosis versus patients during the Omicron wave [75].

Researchers have also investigated the protection provided by an additional vaccine shot or prior infection in relation to Omicron [83,84,85]. According to Deng et al.’s 2023 study, among individuals experiencing SARS-CoV-2 reinfection, 41.77% were asymptomatic, while 51.83% exhibited symptoms [86]. Only a small 0.58% experienced severe symptoms, with a negligible 0.04% progressing to a critical state. Rates of hospitalization, intensive care, or mortality linked to reinfections were reported at 15.48%, 3.58%, and 2.96%, respectively [86]. In comparison to primary infection cases, individuals with reinfections were more commonly affected by milder symptoms (with an odds ratio [OR] of 7.01), and the likelihood of severe symptoms was significantly reduced by 86% (OR = 0.14) [86]. An initial infection, therefore, provided a certain level of immunity against subsequent reinfections. Crucially, reinfections resulted in no further increases in hospitalizations, ICU admissions, or mortality [86].

#### 3.2.1. Transmission

Vaccination or prior infection provides immunity that decreases both the likelihood of spreading the virus and becoming infected. Notably, natural immunity from previous infection plays a more significant role in reducing viral transmission [87]. The primary immunological factor in reducing the chance of spreading the infection to close contacts is natural infection, while the role of vaccination is comparatively minor [87]. However, the impact of vaccination on reducing the rate at which the virus is transmitted remains more consistent over time and is less affected by changes in viral strains compared to its effect on reducing susceptibility to infection, thereby constituting a substantial contribution of vaccines to limiting the spread of SARS-CoV-2 [87].

Other factors affecting transmission include symptom type, such as coughing; the overall health status of the exposed individual; the environment in which the contact occurred (such as home or workplace); and the likelihood of the contact undergoing testing. Vaccination offers protection to both the initial carrier and their contacts, with a more pronounced protective effect observed in the latter, reflecting earlier findings [88]. The diminishing efficacy of vaccination over time and the ability of subsequent variants to better evade immunity when compared to their predecessors underscore the importance of the timing of the most recent vaccination and the variant of concern (VoC) for the level of protection for contacts [88,89]. During the Omicron wave, vaccination within six months did not provide additional protection to contacts, in contrast to non-vaccinated and infection-naïve individuals [88,89]. However, Omicron’s ability to bypass immunity did not reduce the decreased transmission resulting from recent vaccination, suggesting that vaccines continue to lower viral load in Omicron-infected individuals, aligning with findings that vaccination reduces the risk of severe disease for this VoC [90]. Vaccinations administered over six months prior still decreased infectivity, but did not offer protective benefits against contracting the infection for contacts during the Delta wave [87].

#### 3.2.2. Clinical Severity

Despite variations in the level of immunity provided by previous infection, immunization, or a combination of both (hybrid immunity), all three types (natural, vaccination, hybrid) provided over 90% effectiveness in minimizing clinical severity, irrespective of the viral strain involved [91,92,93]. Therefore, the evidence confirms the robust nature of any type of immunity in defending against severe forms of the infection and indicates that when breakthrough infections occur, they are unlikely to result in severe outcomes [83,91]. This is consistent with other studies that suggest that the likelihood of severe manifestations in reinfections is approximately 90% lower than in primary infections, and that the protection provided by vaccination against severe COVID-19 persists beyond its protection against mere infection [83,91].

The probability of developing pneumonia was significantly higher among individuals who had not been vaccinated (65.6% compared to 36.8%), as was the need for oxygen supplementation (29.0% versus 15.7%) compared to their vaccinated counterparts [3]. Vaccinated patients also experienced a significantly shorter duration from the onset of symptoms to hospital discharge compared to those who had not been vaccinated (median of 10 days versus 11 days; ρ < 0.001) [3]. Analyses revealed that vaccination was associated with a decreased risk of pneumonia by approximately 70% and a reduced need for additional oxygen by about 82% [3]. Furthermore, vaccination was linked to a significantly lower risk of both pneumonia and severe disease in the event of a breakthrough infection [3]. Naturally, risk factors and specific virus variants strongly dictate clinical severity as elderly or immunocompromised patients are significantly more likely to develop pneumonia versus healthy controls [3]. Equally, the Delta variant, which mostly targets the lower respiratory system, is reported to increase the mortality risk versus the Omicron variant, which mostly targets the upper respiratory tract [83,84,85,86].

#### 3.2.3. Post-COVID Sequelae

Long COVID-19 (LC) is characterized by a constellation of incapacitating symptoms, often including persistent fatigue and muscle pain, malaise, reduced appetite, and mental impairments [94,95]. Previously published research has demonstrated that there is a significant occurrence of this syndrome in individuals who have had mild-to-moderate COVID-19, despite previous immunization [96]. Advanced age is also recognized as an important factor influencing the severity of the condition, largely due to the gradual decline in immune response as individuals age [96]. Despite uncertainty concerning the occurrence and persistence of specific LC symptoms, patient reports suggest that fatigue can manifest shortly after recovering from COVID-19 [94]. Previously published work has reported that fatigue commonly lingers for six months in patients who have recovered from an initial infection, while immunological disruptions could extend for about eight months following mild-to-moderate COVID-19 infection [97]. In some cases, SARS-CoV-2 may persist in individuals, leading to long-lasting symptoms associated with chronic inflammation and impairment in various organs and tissues [97]. Systematic analysis has notably linked post-infection chronic cognitive impairment with female gender and established an association with the overall clinical severity of the disease, particularly with the presence of respiratory symptoms [98]. However, emerging data suggest that LC might develop regardless of the initial symptom severity [99]. The onset of this chronic illness may be linked to the presence of lingering viral fragments coupled with a persistent systemic immune reaction [100]. Documenting the complex relationship between humoral and cellular immune responses and the immunization status of individuals is vital for preparing relevant therapeutic and vaccination strategies [100]. The immunological research conducted thus far has not identified meaningful immunological discrepancies in LC patients, irrespective of immunization status [94]. Noticeable reductions in IgG and neutralizing antibodies have been observed in participants who had not received a booster vaccine before contracting the infection, indicative of declining immunity [94]. However, notably high antibody levels were still detected at three months post-COVID (PC) [94]. Individuals exposed to BA.5/BA.4 recorded elevated humoral and cellular responses versus patients exposed to other Omicron subtypes [94]. Importantly, higher levels of anti-RBD antibodies were reported after Pfizer-BioNTech immunization in some LC patients [94]. These findings demonstrate that the pathogenesis of LC might involve persistent viral antigens, the reactivation of latent herpesviruses, and ongoing inflammation, potentially correlating with heightened antibody responses [101]. T cell profiling, including memory T cell subsets, has been examined and proposed as pivotal in understanding the differences in disease severity and recovery among COVID-19 patients [101]. The reviewed literature indicates that the numbers of various CD+ T cells are lower in clinically severe cases compared to individuals with non-severe infections [102,103,104]. Nevertheless, no significant disparity was reported regarding T cell concentration levels between healthy controls and LC patients [102,103,104].

According to the World Health Organization, over 10% of infected individuals may develop some form of LC symptomatology, despite previous vaccinations [105]. Although illness rates substantially differ depending on virus subtype (e.g., Delta versus Omicron variant) and other risk factors, the immunological research continues to remain inconclusive. Additionally, other studies have reported that the post-viral symptoms of COVID-19 are no different to an influenza infection in either severity or variety [106]. Therefore, LC remains an actively debated topic among the scientific and medical community globally.

## 4. Conclusions

Despite significant progress in unravelling the underlying immunological mechanisms of hybrid immunity, larger studies that delve into subsets of humoral and cellular immunity need to be explored. Vaccines and prior infection have systematically demonstrated value in reducing disease severity and transmission during reinfection. Nevertheless, post-COVID sequelae remain a largely unexplored area despite significant scientific progress over the last couple of years. Furthermore, due to the volatile scientific nature of the post-COVID sequelae phenomenon, we have witnessed evolving definitions of the disease across major healthcare institutions worldwide and conflicting research findings. As new variants of concern and new vaccines are expected to emerge, ongoing research into the impact of prior infection and immunization in the protection from negative clinical outcomes is crucial to continue [107]. Additionally, the list of risk factors may be expected to evolve and new variants may emerge that may pose an increased risk to specific age groups or patients with specific comorbidities. Guidelines that capture all of these elements of disease management, prevention, and the personalized immunological profiling of COVID-19 patients are fundamental strategies for tackling future pandemics as well.

## Figures and Tables

**Table 1:** Summary of the included studies.

Included Studies	Population Size (N) and Features	Investigated Variables
Zhu X. et al., 2023 (USA)	N = 822; 78% unvaccinated, 6% partly vaccinated, 16% fully vaccinated	Levels of cytokines and chemokines in infected individuals
Wang X. et al., 2022 (USA)	N = 295,691; 98% no prior infection, 5.9% partly vaccinated, 35% fully vaccinated, 19.7% fully vaccinated/boosted	Correlation between immunization, previous infection, and clinical outcomes
Madewell Z.J. et al., 2022 (USA)	N = 135 studies; over 1.3 million participants	Longitudinal assessment by viral strain and vaccination status on household secondary attack rates
Guo L. et al., 2022 (China)	N = 1096; 26.4% moderate COVID-19 disease, 67% severe, 6.7% critical disease	Sustainability and efficacy of humoral and cellular responses in cases recovered from infection after a twelve-month period
Deng J. et al., 2023 (China)	N = 19 studies; 34,375 reinfection cases and 5,264,720 primary infection cases	Susceptibility to severe infection and adverse outcomes following reinfection
Pongkunakorn T. et al., 2022 (Thailand)	N = 292; 158 Long COVID cases and 134 healthy controls	Immunity profile of Long COVID cases versus healthy controls during the Omicron wave
Yorsaeng R. et al., 2023 (Thailand)	N = 4126; 47.6% fully vaccinated, 46.6% fully vaccinated/one booster, 5.8% fully vaccinated/two boosters	Antibody dynamics after immunization or hybrid immunity
Hvidt A.K. et al., 2023 (Canada)	N = 93; 100% unvaccinated initially	Durability of COVID-19-specific immune reaction after infection
Havervall S. et al., 2022 (Sweden)	N = 289 SARS-CoV-2-naïve and N = 118 SARS-CoV-2-recovered	Longitudinal immunological profiling to immunization after infection
Mongin D. et al., 2023 (Switzerland)	N => 50,000 cases; 80.7% unvaccinated and non-infected	Correlation between the secondary attack rate and protective immunity conferred by natural infection and/or immunization
Menni C. et al., 2022 (UK)	N = 620,793; 100% fully vaccinated	Primary vaccine series effectiveness and waning
Ontañón J. et al., 2021 (Spain)	N = 63; 33 prior infection and 30 infection-naive	Persistence and dynamics of antibody-mediated immune reaction after full immunization
Seo W.J. et al., 2022 (Republic of Korea)	N = 387; 204 fully vaccinated and 183 unvaccinated	Association of prior immunization and clinical outcomes
Altarawneh H.N. et al., 2023 (Qatar)	N = 239,120 PCR-positive samples	Impacts of past infection, immunization, and hybrid immunity on symptomatic infections by different variants

## Data Availability

No new data were created or analyzed in this study. Data sharing is not applicable to this article.
